# Effect of Ag Nanoparticles on Denitrification and Microbial Community in a Paddy Soil

**DOI:** 10.3389/fmicb.2021.785439

**Published:** 2021-12-22

**Authors:** Xiao Zhang, Di Dang, Lingsi Zheng, Lingyu Wu, Yu Wu, Haoruo Li, Yongjie Yu

**Affiliations:** ^1^Key Laboratory of Agrometeorology of Jiangsu Province, Nanjing University of Information Science and Technology, Nanjing, China; ^2^Key Laboratory of Karst Dynamics, MNR and Guangxi, Institute of Karst Geology, Chinese Academy of Geological Sciences, Guilin, China

**Keywords:** Ag nanoparticles, paddy soil, nitrous oxide, denitrifying genes, high-throughput sequencing

## Abstract

The extensive application of Ag nanoparticles (AgNPs) in industry, agriculture, and food processing areas increases the possibility of its release and accumulation to agroecosystem, but the effects of AgNPs to denitrification and the microbial community in paddy ecosystems are still poorly studied. In this study, microcosmic simulation experiments were established to investigate the response of soil denitrification to different levels of AgNPs (i.e., 0.1, 1, 10, and 50 mg/kg) in a paddy soil. Real-time quantitative PCR and high-throughput sequencing were conducted to reveal the microbial mechanism of the nanometer effect. The results showed that, though 0.1–10 mg/kg AgNPs had no significant effects on denitrification rate and N_2_O emission rate compared to CK and bulk Ag treatments, 50 mg/kg AgNPs significantly stimulated more than 60% increase of denitrification rate and N_2_O emission rate on the 3rd day (*P* < 0.05). Real-time quantitative PCR revealed that 50 mg/kg AgNPs significantly decreased the abundance of 16S bacterial rRNA gene, *nirS*/*nirK, cnorB*, and *nosZ* genes, but it did not change the *narG* gene abundance. The correlation analysis further revealed that the cumulative N_2_O emission was positively correlated with the ratio of all the five tested denitrifying genes to bacterial 16S rRNA gene (*P* < 0.05), indicating that the tolerance of *narG* gene to AgNPs was the key factor of the increase in denitrification in the studied soil. High-throughput sequencing showed that only the 50-mg/kg-AgNP treatment significantly changed the microbial community composition compared to bulk Ag and CK treatments. The response of microbial phylotypes to AgNPs suggested that the most critical bacteria which drove the stimulation of 50 mg/kg AgNPs on N_2_O emission were Firmicutes and β-proteobacteria, such as Clotridiales, Burkholderiales, and Anaerolineales. This study revealed the effects of AgNPs to denitrification in a paddy ecosystem and could provide a scientific basis for understanding of the environmental and toxicological effects of Ag nanomaterials.

## Introduction

Nanomaterials were widely applied in a multitude of industries due to their advantages in special physiochemical properties, quantum size effect, and specific surface activity (Maynard et al., 2006; Gan and Li, 2012; Goldberg, 2019; He et al., 2019; Nasrollahzadeh et al., 2021). The global consumption of nanoparticles is predicted to be ~900 tons per year by 2030 (Olteanu et al., 2017; Yaqoob et al., 2020). Among nanoparticles like Au, ZnO, CuO, TiO_2_, and Fe_2_O_3_, silver nanoparticle (AgNPs) is a typically widely used nanomaterial due to its significant advantages in electrical conductivity, antibiosis, and localized surface plasmon resonance properties (Salleh et al., 2020). These vital properties of AgNP make it not only prominent in the industries of photonics, microelectronics, and catalysis but also popular in healthcare, environmental preservation, and bactericide in agriculture (Hamad et al., 2020). With the application of AgNPs spanning across diverse fields, it inevitably increases the possibility of its release and accumulation in natural environments (Whitley et al., 2013; Prosposito et al., 2020). It has been reported to be found in various ecosystems, such as oceans, freshwater, wetland, and soils (Lead and Wilkinson, 2006; Wilson et al., 2008). According to the modeling results, the emission of nanomaterials in a soil environment is much more abundant than that in aquatic and atmospheric environments (Gottschalk et al., 2009; Sun et al., 2014). Although the application of AgNPs in agriculture is a prospective area (Pradas Del Real et al., 2016), the influence of AgNPs on soil ecosystem is still not fully understood.

Paddy soils support the staple diet for nearly 50% of the global population (according to FAOSTAT in 2019). The negative effects of engineered nanomaterials on the paddy ecosystem gained great concerns in recent years. Long-term anaerobic and low-oxygen conditions make soil denitrification the main nitrogen cycling process (Cai et al., 1997). Since the oxidation of AgNPs to silver ions inevitably changes the electron transport chain processes, soil denitrification, a series of biochemical oxidation–reduction reactions, might be influenced by the released AgNPs in a paddy ecosystem. Furthermore, the potentially influenced soil N_2_O emission in denitrification is also an important content in the risk assessment of the released AgNPs. It has been reported that 1 mg/L AgNPs inhibited the nitrogen cycling processes in a sludge treatment system (Liang et al., 2010). Zheng et al. (2017) demonstrated that 2 mg/L AgNPs significantly changed the denitrification processes and stimulated N_2_O emission in aquatic ecosystems. So far, the environmental risk assessment of AgNPs is still at the primary stage, and lots of investigations concentrate on the aquatic ecosystem. Thus, the knowledge gap concerning the influence of AgNPs on denitrification in paddy ecosystems still remains.

Soil microorganism, which drives the conversion of organic carbon and nutrient cycling, is considered as one of the most important sensitive indicators in terrestrial ecosystems. It has been well-known that a microbial community could dynamically regulate them to the change of environmental factors (Delgado-Baquerizo et al., 2016; Zheng et al., 2017), so it may be perceptive to the exposure of AgNPs. It has been demonstrated that microbial community and diversity can be significantly influenced by AgNPs (Cao et al., 2017; Samarajeewa et al., 2017; Zhang et al., 2020). Kumar et al. (2011) demonstrated that the amendment of 50 mg kg^−1^ AgNPs altered the soil bacterial community structures in arctic soil. Wang et al. (2017) also reported that the AgNP concentration of more than 20 mg/kg seriously inhibited the growth of soil microbes in suburban vegetable soils. Yang et al. (2013) demonstrated that AgNPs significantly inhibit the abundance and activity of *Nitrosomonas europaea* in sewage treatment systems. It can be inferred that AgNPs could show exhibited toxicity to soil-denitrifying communities when it is exposed to a paddy ecosystem. Although there is rich literature examining the responses of microbial community and function to AgNPs (Kent et al., 2014; Guo et al., 2019; Huang et al., 2019a), few studies investigate how AgNPs affect denitrifying communities in paddy soil ecosystems. More specifically, the underlying mechanistic details of the denitrifying community and the phylogenetic structure in response to different AgNP concentrations in paddy soils are seldom available.

Here we applied different concentrations of AgNPs to paddy soil at the microcosmic scale under laboratory conditions in order to simulate the scenarios of nanomaterial exposed to a paddy ecosystem. A combination of real-time quantitative PCR and high-throughput sequencing technologies was conducted to reveal the microbial mechanism underneath the response of denitrification to AgNPs. This study aims to enhance our understanding of the impacts that AgNPs bring to paddy soil and provide a theoretical and scientific basis for better evaluating the ecological effects of nanomaterials in agriculture.

## Materials and Methods

### Soils and Nanoparticles

The tested paddy soil samples were from Yingtan, Jiangxi Province (116°55′30” E, 28°15′20” N). Surface (0–15 cm) soil was obtained after rice paddy harvest. Fresh soil was sieved through a 2-mm mesh, with visible roots, stones, and macro-fauna removed. One subsample was air-dried for chemical analyses. One fresh subsample was stored at 4°C before analysis, whereas the residual subsample was immediately stored at −80°C for molecular analysis. The pH of the tested soil was 5.01, soil organic carbon was 14.58 g/kg, total nitrogen was 1.09 g/kg, ${\text{NO}}_{3}^{-}$-N was 1.2 mg/kg, and ${\text{NH}}_{4}^{+}$-N was 4.4 mg/kg.

AgNP was synthesized with a chemical reduction method described according to Feng et al. (2013). In detail, firstly, a precursor of 0.01 M AgNO_3_ solution and 1% PVP (molecular weight = 40,000) were mixed at a molar ratio of 1:1.45 to get an optimum stabilizing effect of PVP with a minimum particle size. Then, AgNO_3_ solution was added drop by drop to a stirred and preheated (60°C) solution with the components of 0.01 M NaOH and 0.02 M glucose (about 30 drops per minute). The pH range throughout the reaction was kept in between 8.5 and 9.0. Stirring of the solution lasted until 10 min after all the AgNO_3_ solution was added. Then, the obtained brownish-silver colloid was centrifuged and washed with distilled water several times until no nitrate could be traced. The precipitated AgNPs were separated by centrifugation at 4,000 × *g* for 10 min and subsequently allowed to air-dry in a desiccator. The dry silver particles were re-dispersed and washed with deionized water and dispersed by ultrasonic oscillation to obtain a stable and uniform aqueous solution. The average size of the synthesized AgNPs in this study was 20.5 ± 3.5 nm. The zeta potential (ζ) of AgNPs was −22.9 ± 1.1 mV. Bulk Ag powder was purchased from Sinopharm Chemical Reagent Co., Ltd., China. The particle size of the bulk Ag powder was ~5.0 μm.

### Experimental Design

For each soil sample, a series of 250-ml Erlenmeyer flask was prepared with 30 g soil (oven-dry basis) and then divided into three groups, i.e., AgNPs, bulk Ag, and control (without Ag particle amendment). Four levels of AgNPs or bulk Ag were applied to each soil sample with the concentrations of 0.1 (R), 1 (L), 10 (M), and 50 mg kg^−1^ (H), which were commonly used in studies of the effects of AgNPs on the ecosystem (Kumar et al., 2011; Zheng et al., 2017). Both AgNPs and bulk Ag were dispersed with 20-min sonication at 600 W; then, the suspensions were added drop by drop to the tested soils to homogenize the Ag particles and soil samples. A certain amount of sterile water was added to each flask to make the ratio of water to soil at 1:1. Silicone sealant was used around the plug to ensure anaerobic conditions. The flasks were connected to a multiport vacuum manifold, which allowed 20 flasks to be simultaneously vacuumed and flushed with oxygen-free N_2_ gas. The procedure was repeated three times (each for 10 min) to create an anaerobic headspace. Following equilibration at atmospheric pressure, the flasks were incubated at 25°C in the dark. A certain amount of sterile water was injected in order to ensure moisture during the whole incubation process. Sampling was performed on the 1st, 3rd, 7th, 14th, and 28th day. The denitrification rate was measured by acetylene inhibition method (Yu et al., 2013). The N_2_O concentration was determined using a 2-mm ID stainless steel column, 3 m in length and packed with Porapak Q (80/100 mesh), and an Agilent 7890 gas chromatograph fitted with an electron capture detector set at 300°C.

### Molecular Methods

Soil total DNA was extracted from 0.5 g fresh soil using the FastDNA^®^ SPIN Kit for soil (MP Biomedicals, Santa Ana, CA, USA) according to the instructions of the manufacturer. The quality and quantity of DNA were checked using a NanoDrop spectrophotometer (NanoDrop Technologies Inc., Wilmington, DE, USA). The soil DNA was stored under −20°C before using.

Quantitative real-time PCR was performed on the target denitrifying genes, i.e., *narG, nirS, nirK, cnorB*, and *nosZ* genes, and bacterial 16S rRNA gene in triplicate according to Yu et al. (2013) in a CFX96 Real-Time system (Bio-Rad, CA, USA). Standard curves were created using 10-fold dilution series of linearized plasmids with target genes inserted. The PCR mixtures for each gene contained 1.0 μl DNA template, 12.5 μl of SYBR^®^ *PremixExTaq*^TM^ (TaKaRa, Dalian, China), and 100 nM of each primer. Thermocycling consisted of 95°C for 5 min, followed by 40 cycles of 95°C for 10 s and annealing for 30 s, and 72°C for 60 s. The annealing temperature was set according to our previous study (Yu et al., 2013). The amplification efficiency was controlled in the range of 90–110%; the standard curve coefficient correlations were between 0.97 and 0.99.

High-throughput sequencing of the bacterial 16S rRNA gene was performed according to our previous study (Yu et al., 2019) on Miseq platform (Illumina Inc., CA, USA). In detail, the primer set 519F and 907R was used to amplify ~400 bp of bacterial 16S rRNA gene fragments. The oligonucleotide sequences included a 5-bp barcode fused to the forward primer as follows: barcode + forward primer. PCR was carried out in 50-μl reaction mixtures with the following components: 4 μl (initial 2.5 mM each) of deoxynucleoside triphosphates, 2 μl (initial 10 mM each) of forward and reverse primers, 2 U of Taq DNA polymerase with 0.4 μl (TaKaRa, Dalian, China), and 1 μl of template containing ~50 ng of genomic community DNA as a template. Furthermore, 95°C for 5 min and 25 cycles of 95°C for 45 s, 56°C for 45 s, and 72°C for 60 s were performed, with a final extension at 72°C for 7 min. The purified barcoded PCR products from all of the samples were normalized in equimolar amounts, then prepared using TruSeq™ DNA Sample Prep LT Kit, and sequenced using MiSeq Reagent Kit v3 (600-cycles-PE) following the protocols of the manufacturer. Moreover, 10% PhiX control library was added to the tested DNA sample before sequencing. The obtained raw sequencing data were uploaded to NCBI Sequence Read Archive database with the accession number of PRJNA768985.

### Statistical Analysis

The analysis of bacterial sequencing data was performed with Quantitative Insights Into Microbial Ecology (QIIME) 1.9.1 pipeline (Caporaso et al., 2010). Briefly, the sequences were binned into OTUs using 97% identity threshold, and the most abundant sequence from each OTU was selected as a representative sequence for that OTU. Taxonomy was assigned to bacterial OTUs against a subset of the Greengene 13.8 database. Usearch was used to remove chimera and aligned OTU representative sequences (Edgar, 2013). The weighted pairwise UniFrac distances (Ley et al., 2005) were calculated for community comparisons using *beta_diversity_through_plots.py* in QIIME. The one-way analysis of variance (ANOVA), permutational multivariate analysis of variance (PERMANOVA), and heat map analysis were calculated with *vegan, ggplot2*, and *reshape2* packages in R software (Fine and Kembel, 2011). A correlation analysis was quantified using analysis of variance. The responses of bacterial species to different treatments were visualized with STAMP (Parks et al., 2014). Differences at *P* < 0.05 were considered statistically significant.

## Results

### Effect of AgNPs on Soil Denitrification and N_2_O Emission Rate

**Figure 1:**
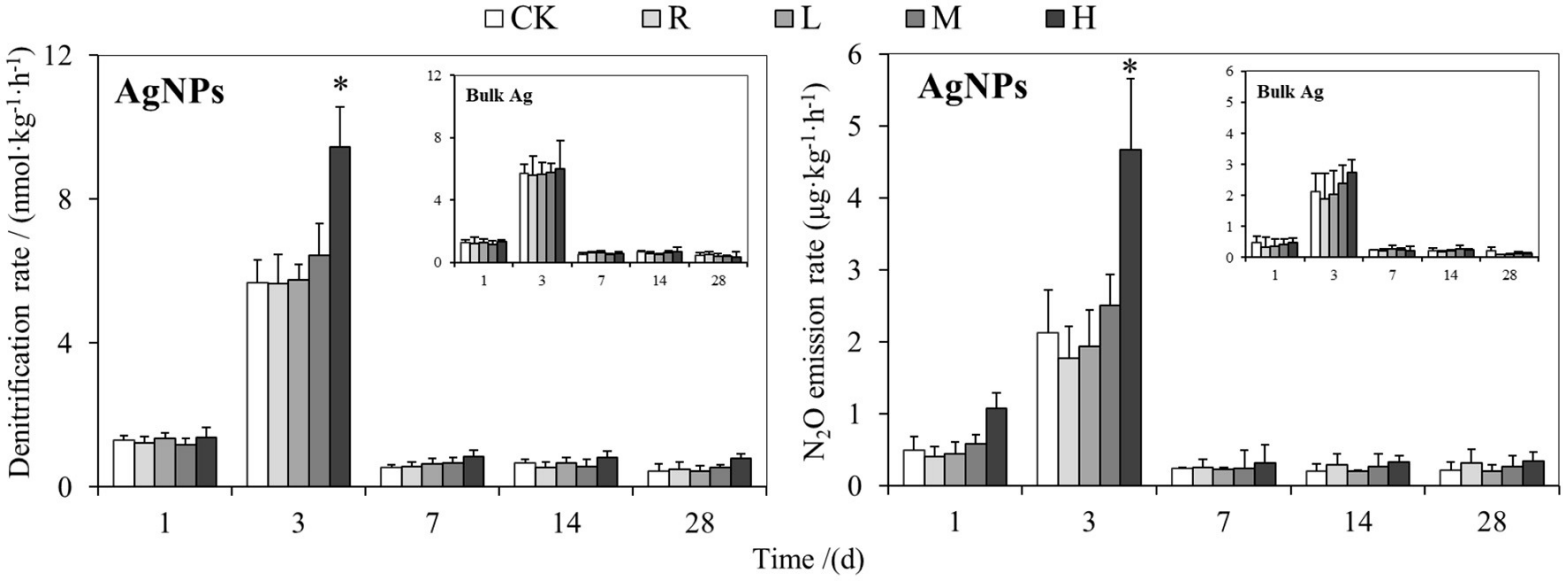
The effects of AgNPs on denitrification and N_2_O emission rate in a paddy soil. R, L, M, and H represent 0.1, 1, 10, and 50 mg/kg, respectively. CK indicates the treatments without Ag particle amendment. Asterisks above the bars represent a significant difference at the 0.05 level.

The responses of soil denitrification and N_2_O emission rate to different concentrations of AgNPs are shown in Figure 1. The peak value of soil denitrification rate was 5.68 ± 0.63 nmol kg^−1^ h^−1^ at day 3 in the CK treatments; then, it decreased to a range between 0.44 and 0.68 nmol kg^−1^ h^−1^ in the tested paddy soil. The N_2_O emission rate reached a peak value of 2.13 ± 0.60 μg kg^−1^ h^−1^ at day 3, and then it changed between 0.01 and 0.02 μg kg^−1^ h^−1^. The peak values of denitrification and N_2_O emission rate in AgNP treatment were also observed at day 3. Furthermore, 50 mg/kg (H) AgNPs significantly increased both the denitrification and N_2_O emission rate at day 3 (*P* < 0.05), but it did not significantly change the denitrification and N_2_O emission rate at all the other incubation times (*P* > 0.05). The other three concentrations, 0.1 (R), 1 (L), and 10 mg/kg (M), of AgNPs did not significantly influence the soil denitrification and N_2_O emission rate at the same incubation time (*P* > 0.05). No significant difference of denitrification and N_2_O emission rate was observed between bulk Ag treatment and CK at the same incubation time (*P* > 0.05).

### Effect of AgNPs on Bacterial and Denitrifying Genes

**Figure 2:**
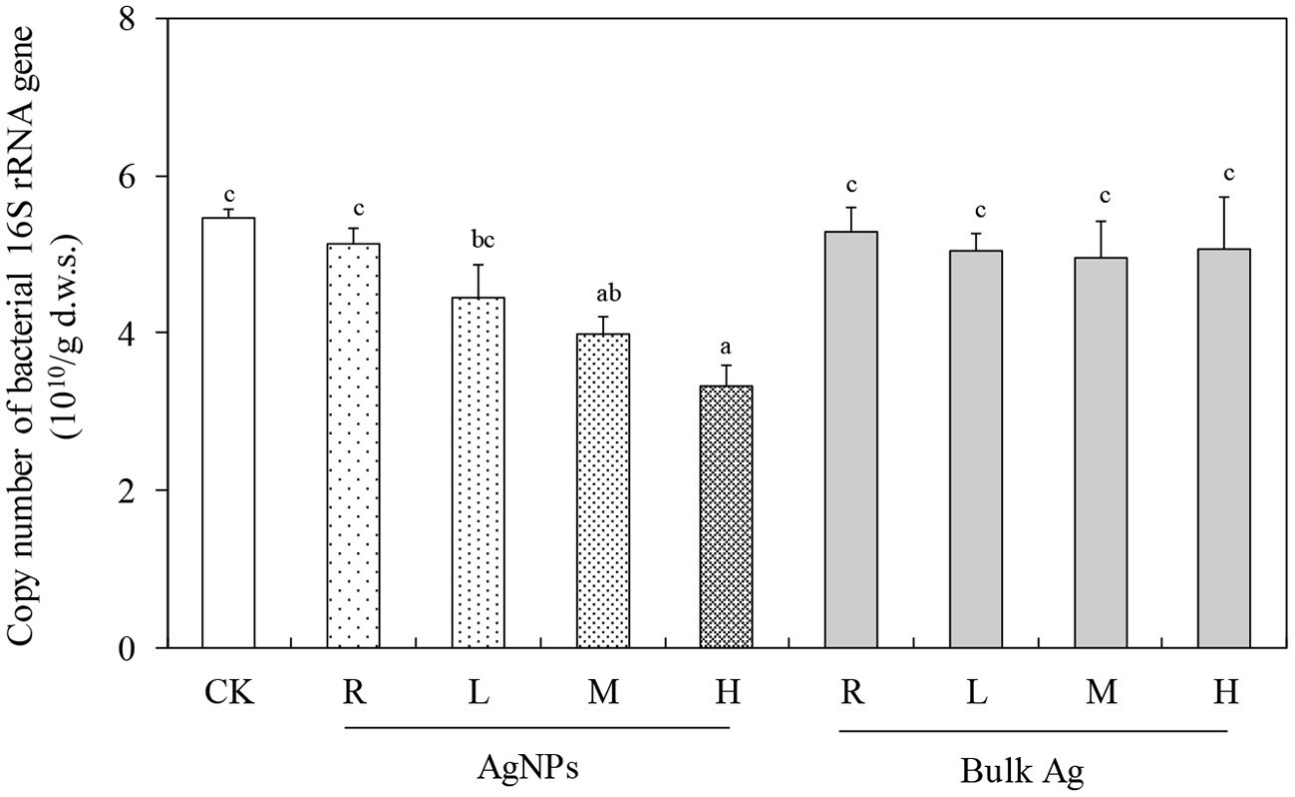
The effects of AgNPs on bacterial 16S rRNA gene abundance in a paddy soil. R, L, M, and H represent 0.1, 1, 10, and 50 mg/kg, respectively. CK indicates the treatments without Ag particle amendment. Lowercase letters above the bars represent a significant difference at the 0.05 level.

The response of bacterial 16S rRNA gene abundance to different concentrations of AgNPs is shown in Figure 2. The copy number of bacterial 16S rRNA gene was 5.46 ± 0.11 × 10^10^ copies g^−1^ d.w.s. in the CK treatment. A concentration effect was observed in different AgNP treatments, that is, the bacterial abundance decreased with the increase in concentration of AgNPs in the tested paddy soil. In detail, 0.1 (R) and 1 mg/kg (L) AgNPs showed a decreasing trend compared to CK, though there was no significant difference between them (*P* > 0.05). Both 10 (M) and 50 mg/kg (H) AgNPs significantly decreased the soil bacterial abundance compared to CK (*P* < 0.05). In addition, 50 mg/kg (H) AgNPs had the lowest abundance of soil bacteria (2.31 × 10^10^ copies g^−1^ d.w.s.) and decreased by about 57.7% of that of CK. No significant difference of bacterial abundance was observed between bulk Ag treatment and CK (*P* > 0.05).

**Figure 3:**
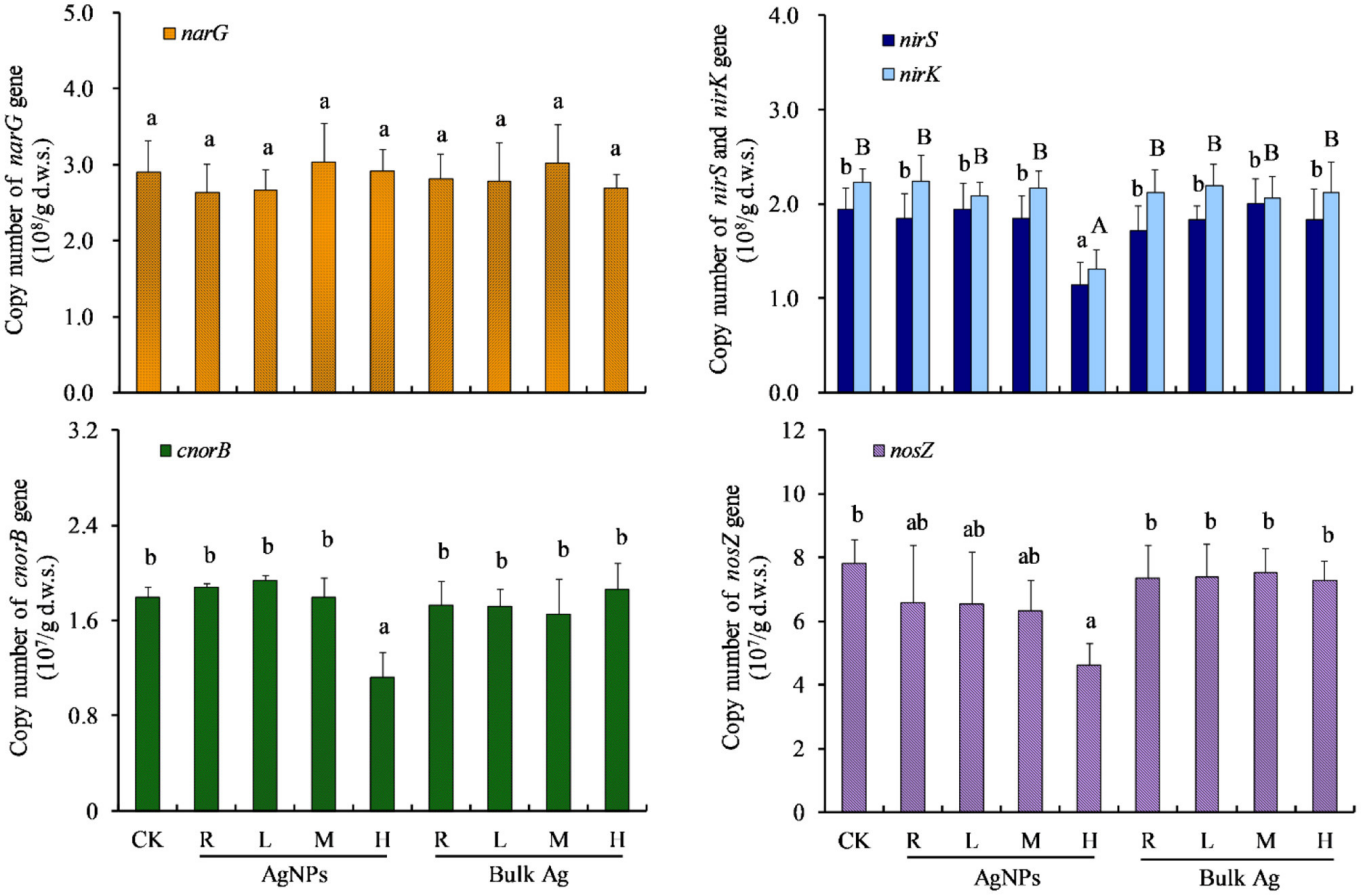
The effects of AgNPs on denitrification functional gene abundance in a paddy soil. R, L, M, and H represent 0.1, 1, 10, and 50 mg/kg, respectively. CK indicates the treatments without Ag particle amendment. Different letters above the bars represent a significant difference at the 0.05 level.

The abundance of *narG, nirS, nirK, cnorB*, and *nosZ* denitrifying genes in CK were 2.90 × 10^8^, 1.94 × 10^8^, 2.23 × 10^8^, 1.80 × 10^7^, and 7.81 × 10^7^ copies g^−1^ d.w.s., respectively (Figure 3). Moreover, 0.1 (R), 1 (L), and 10 mg/kg (M) AgNPs did not significantly change the *nirS, nirK, cnorB*, and *nosZ* genes (*P* > 0.05). Furthermore, 50 mg/kg AgNPs also showed no significant difference in *narG* gene abundance (*P* > 0.05), but it significantly decreased the abundance of *nirS, nirK, cnorB*, and *nosZ* genes (*P* < 0.05). No significant difference of denitrifying gene abundance was observed between bulk Ag treatment and CK (*P* > 0.05).

**Figure 4:**
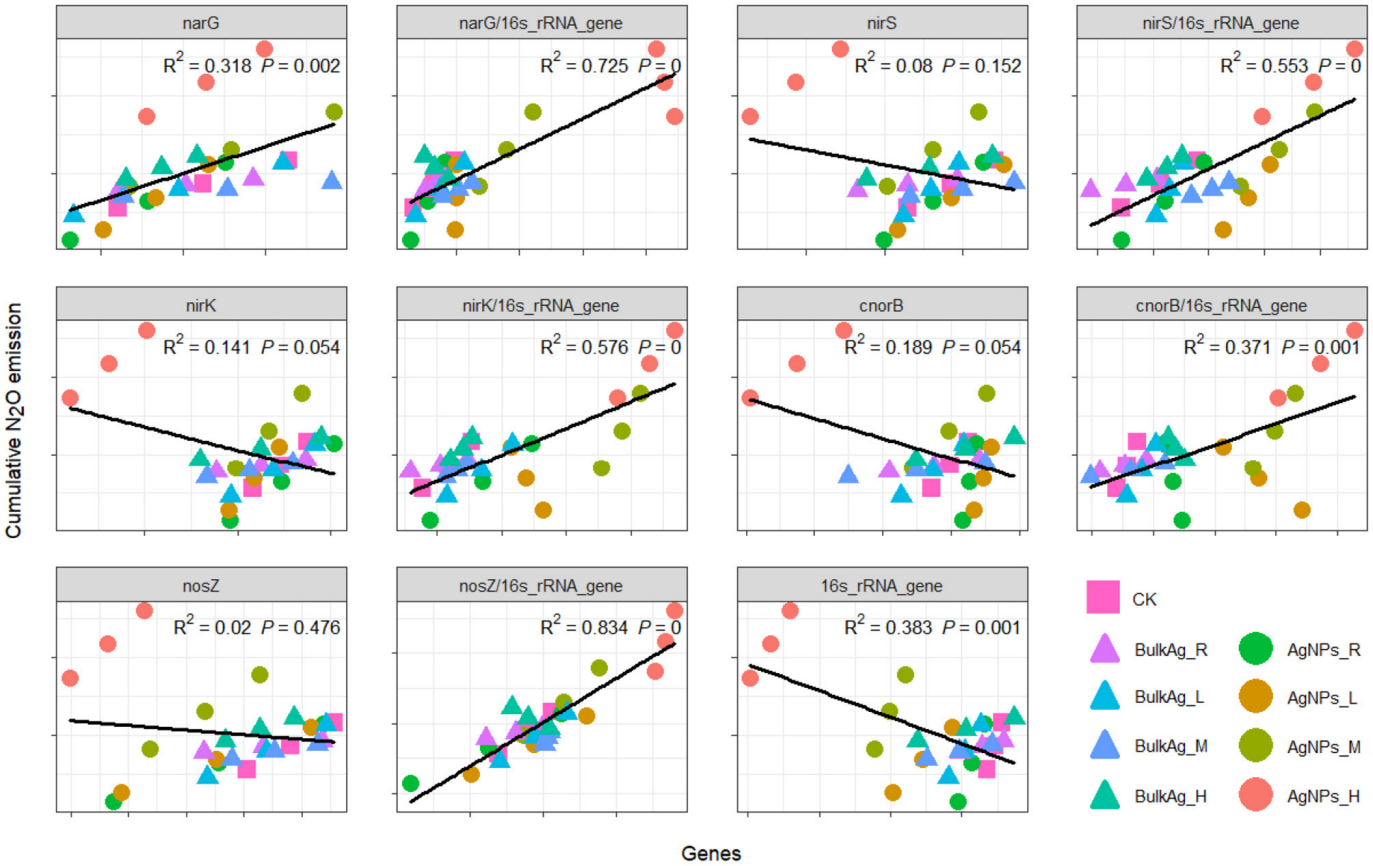
Correlation between cumulative N_2_O emission and denitrifying genes under different application levels of AgNPs in a paddy soil. R, L, M, and H represent 0.1, 1, 10, and 50 mg/kg, respectively. CK indicates the treatments without Ag particle amendment.

To unravel the key microbial factor/s driving the denitrification process, correlation analyses were performed between cumulative N_2_O emission and the abundance of microbial genes in the tested paddy soil (Figure 4). The cumulative N_2_O emission was found to be positively significantly correlated with soil *narG* gene abundance (*R* = 0.564, *P* < 0.05), but it was not significantly correlated with *nirS, nirK, cnorB*, and *nosZ* genes (*P* > 0.05). A negatively significant relationship was observed between cumulative N_2_O emission and bacterial 16S rRNA gene (*R* = −0.619, *P* < 0.05). Further analyses showed that soil cumulative N_2_O emission was positively related with the ratio of all the five denitrifying genes to bacterial 16S rRNA gene copies (*P* ≤ 0.001).

### Effect of AgNPs on Microbial Community

**Figure 5:**
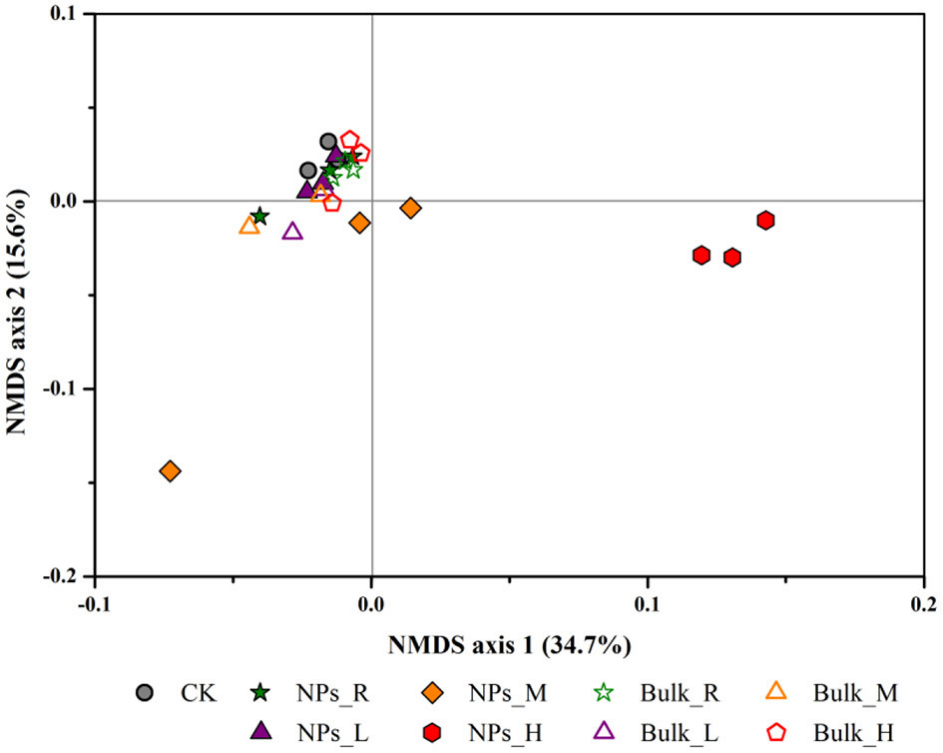
Bacterial community compositional structure as indicated by a non-metric multi-dimensional scaling plot of the weighted pairwise UniFrac community distances under different concentrations of AgNPs in a paddy soil. R, L, M, and H represent 0.1, 1, 10, and 50 mg/kg, respectively. CK indicates the treatments without Ag particle amendment.

To reveal the response of microbial community to AgNPs, high-throughput sequencing was performed on Miseq platform. In total, we obtained 435,827 sequences in the tested soils and 14,647–26,129 sequences per sample. Taxonomy against gene bank showed 99.3% bacteria, 34.60% Proteobacteria, 14.90% Acidobacteria, 11.70% Chloroflexi, 8.00% Actinobacteria, and 7.90% Firmicutes. According to the weighted distance of UniFrac obtained by non-metric multi-dimensional scaling (NMDS), the distribution of community composition of methanogenic archaea under different treatments is shown in Figure 5. NMDS showed that the soil bacterial community composition differed with the increase of AgNP concentration (Figure 5). The PERMANOVA result found no significant difference between CK and 0.1 (R), 1 (L), and 10 mg/kg (M) AgNP treatments. The bacterial community composition of 50 mg/kg (H) AgNP treatments was significantly different from all the other treatments (*P* < 0.01). The bulk Ag treatments did not significantly change the bacterial community composition compared to the CK treatments (*P* > 0.05).

**Figure 6:**
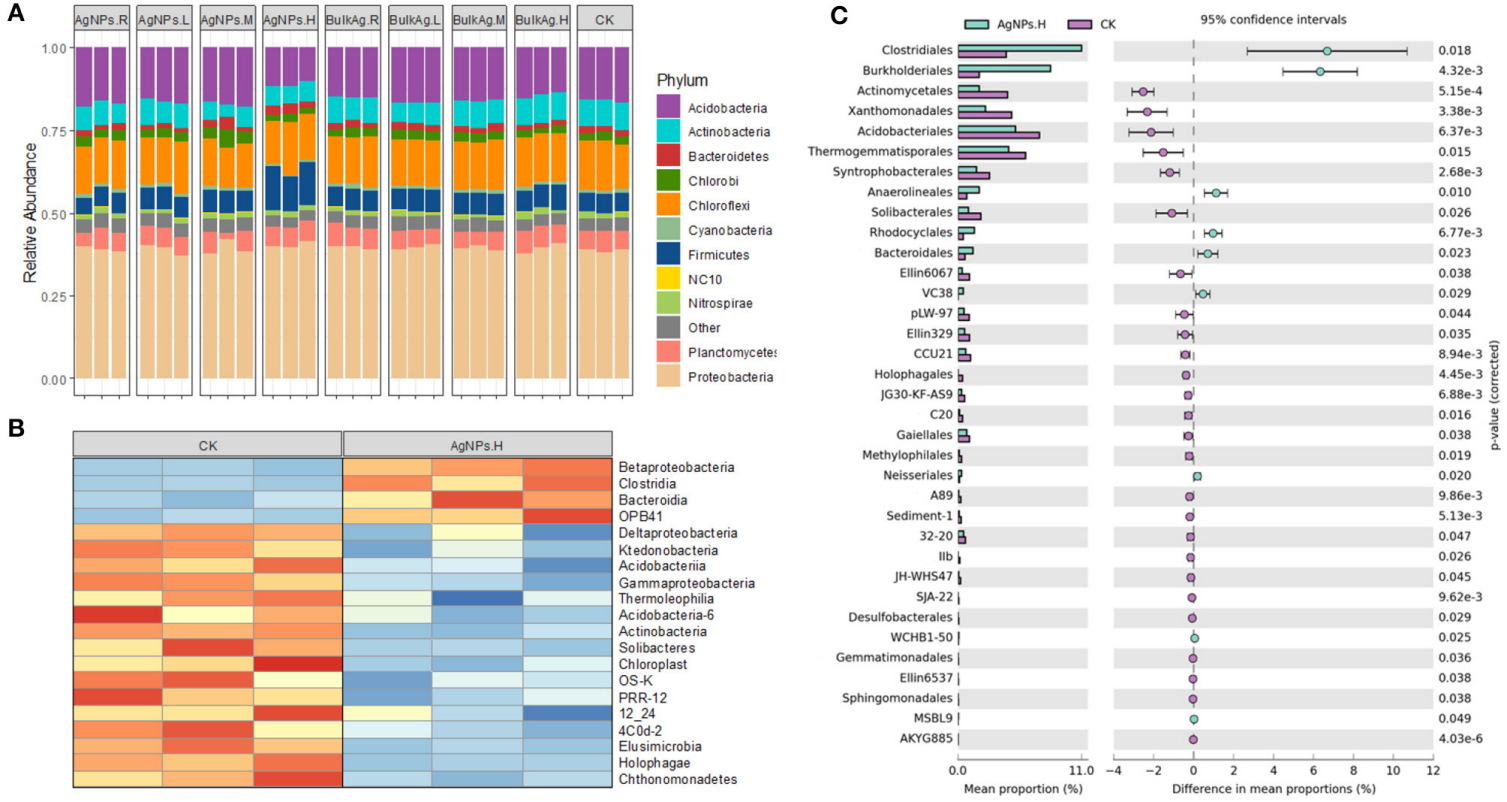
The bacterial community variation characteristic under different concentrations of AgNPs in a paddy soil. **(A)** The relative abundance of soil bacteria at the phylum level. **(B)** The heat map of soil bacteria at the class level. **(C)** The response ratio of soil bacteria at the order level. R, L, M, and H represent 0.1, 1, 10, and 50 mg/kg, respectively. CK indicates the treatments without Ag particle amendment.

To unravel the key microbial phylotype/phylotypes related to the influence of AgNPs on soil denitrification, bacteria at different taxonomy levels were statistically analyzed between different treatments in the tested paddy soil (Figure 6). At the phylum level, 50 mg/kg (H) AgNPs decreased Acidobacteria, Actinobacteria, Elusimicrobia, and Cyanobacteria, while it increased Firmicutes and WS5 phyla (Figure 6A). Moreover, 0.1 (R), 1 (L), and 10 mg/kg (M) AgNP treatments rarely changed the bacterial phyla compared to that of CK treatments (Figure 6A). At the class level, 50 mg/kg (H) AgNPs decreased Deltaproteobacteria, Gammaproteobacteria, and Ktedonobacteria, while it increased Betaproteobacteria, Clostridia, Bacterodia, and OPB41 (Figure 6B). At the order level, the response ratio analyses showed that 50 mg/kg (H) AgNPs significantly decreased most of the bacteria orders but increased Clotridiales, Burkholderiales, and Anaerolineales (*P* < 0.05).

## Discussion

The highly commercialized AgNPs could be enriched, migrated, or transformed in the soil ecosystem due to its remarkable characteristics, such as specific surface activity and active physiochemical properties (Wilson et al., 2008; Sun et al., 2014). The potential influences of AgNPs on soil ecosystems are highly concerning (He et al., 2019; Lowry et al., 2019). In this study, a series of microcosm experiments was conducted to simulate the scenario of a paddy ecosystem exposed under different concentrations of AgNP. The responses of soil denitrification and microbial community were investigated by anaerobic incubation and molecular methods.

### Responses of Soil Denitrification Rate to AgNPs

The oxidation process of AgNPs to silver ions in soils inevitably changes the electron transport chain processes in soils (Huang et al., 2019b) and subsequently influences nitrogen cycling that is substantially biochemical reduction and oxidation reactions. Previous studies found that 1 mg/kg AgNPs could inhibit the nitrification bacteria (Choi and Hu, 2008; Radniecki et al., 2011). Grün et al. (2018) demonstrated that a 1-year application of low-concentration AgNPs (0.01–1 mg/kg) could cause a negative influence on soil Azotobacter and ammonia bacteria. In this study, we found that neither denitrification nor N_2_O emission rate was significantly influenced by the 0.1–10 mg/kg AgNPs (*P* > 0.05). This phenomenon mainly resulted from the complex environment in paddy soils, which is radically different from laboratory culture and aquatic environments. When AgNPs (<10 mg/kg) was exposed to soils, it could be captured by colloidal solids, clay minerals, iron oxides, and manganese oxides in soils (Alvarez and Cervantes, 2012; Vandevoort et al., 2014), and then the toxicity of AgNPs might be buffered. This finding is consistent with the previous study, in which 1 mg/kg AgNP did not change the denitrification in a sediment environment (Liu et al., 2018).

A high concentration up to 50 mg/kg AgNPs significantly changed both the denitrification and N_2_O emission rate in this study (*P* < 0.05). We found that bulk Ag treatments had no influences on denitrification and N_2_O emission rate compared to CK treatment, indicating that the influence of high-concentration AgNPs on soil denitrification resulted from a nanometer effect. Interestingly, the effects of 50 mg/kg AgNPs on soil denitrification did not show an inhibition effect; it was a stimulating effect. The possible reason for this phenomenon might be that some metabolic activities or associated microbes in the tested paddy soil were stimulated by high concentration of AgNPs, which is named as the hormesis effect (Grün et al., 2018). To reveal the specific microbial mechanism in the hormesis effect of 50 mg/kg AgNPs on the tested paddy soil, the abundance of the denitrifying genes and bacterial gene were detected by real-time PCR. It was found that 50 mg/kg AgNPs decreased the abundance of *nirS, nirK, cnorB*, and *nosZ* and bacterial gene copies in soil, but did not significantly changed the abundance of *narG* gene (*P* < 0.05), which is considered as the indicator of nitrate reductase (Yu et al., 2013). Further correlation analyses revealed that, though cumulative N_2_O emission was negatively correlated with bacterial abundance, it is positively correlated with the ratio of *narG* gene to the bacterial gene and with the ratio of the other four denitrifying genes to the bacterial gene. These results indicated that the *narG* gene could tolerate the toxicity of AgNPs in the case of all soil bacteria that were inhibited by 50 mg/kg AgNPs. In this situation, soil denitrifiers with *narG* gene could utilize the spare nitrogen and carbon resources that were occupied by the inhibited bacteria (Yang et al., 2013), and then more ${\text{NO}}_{3}^{-}$ was reduced to ${\text{NO}}_{2}^{-}$. High ${\text{NO}}_{2}^{-}$ concentrations subsequently promote denitrification processes and N_2_O production (Cai et al., 1997). A positive correlation between cumulative N_2_O emission and the ratio of *nirS, nirK, cnorB*, and *nosZ* genes to the bacterial gene indicated that the denitrifying community could be more active than the other bacteria. In addition, it was reported that chemical process (e.g., chemodenitrification) is important for enhancing soil N_2_O production (Chen et al., 2018; Liu et al., 2019). In this study, the *narG* gene insensitive to AgNPs accumulated ${\text{NO}}_{2}^{-}$ in the tested soil, which could promote the chemodenitrification processes. As a result, the soil denitrification and N_2_O emission rates were significantly higher in 50 mg/kg AgNPs than that of CK.

### Key Microbial Phylotypes in Paddy Soil Exposed to AgNPs

In paddy soils, microbes drive the denitrification processes in the anaerobic situation. The influence of high-concentration AgNPs on denitrification inevitably reflects in the change of some key phylotypes in soils (Yu et al., 2013; Grün et al., 2018). High-throughput sequencing showed a concentration effect of AgNPs on the microbial community composition, that is, the increase in the AgNP concentration tended to distribute in the microbial community. The difference of the distribution was found significant in the 50-mg/kg-AgNP treatments. This result is consistent with the previous study on the influence of AgNPs on soil fungal community (Feng et al., 2013). Further analyses showed that 50 mg/kg AgNPs decreased most of the bacteria phyla, such as Acidobacteria, Actinobacteria, Elusimicrobia, and Cyanobacteria. This result corresponded to the toxicity of AgNPs on soil bacteria (Vandevoort et al., 2014). It has been demonstrated that the released AgNPs can damage the cell membrane and affect cellular viability by inducing the production of intracellular reactive oxygen species and free radicals (Hansch and Emmerling, 2010; Zhou, 2015), so the abundance of bacterial 16S rRNA gene was decreased in the 50-mg/kg-AgNP treatments. The denitrifying species in soils are mainly classified as Firmicutes and Proteobacteria (Throback et al., 2007). In this study, Clotridiales, Burkholderiales, and Anaerolineales, which are Firmicutes and β-proteobacteria, were significantly decreased by the 50-mg/kg-AgNP treatment. This phenomenon suggested that these potentially functional denitrifiers can tolerate the toxicity of AgNPs. We also observed that the 50-mg/kg-AgNP treatment increased Clotridiales and Burkholderiales. The thick cell walls of Clotridiales (Lee et al., 2013) and the good toxicity tolerance of Burkholderiales (Lee et al., 2013) could make the paddy soil possess higher denitrification than the CK treatments. Overall, Clotridiales, Burkholderiales, and Anaerolineales could be the key microbial species in the stimulation of 50 mg/kg AgNP on denitrification in the tested paddy soil.

## Conclusion

The results of the present study demonstrate the influence of AgNPs on denitrification and microbial communities in a paddy soil. Here we show that soil denitrification and N_2_O emission were stimulated by 50 mg/kg AgNPs, while it is not susceptible to 0.1–10 mg/kg AgNPs. The tolerance of soil *narG* gene to 50 mg/kg AgNPs is the key factor which promotes denitrification in 50-mg/kg-AgNP-treated soils. The key bacterial phylotypes inducing high denitrification in 50-mg/kg-AgNP treatments could be Clotridiales, Burkholderiales, Anaerolineales, Clotridiales, and Burkholderiales in the tested paddy soil. These results provide valuable information for risk assessment of AgNPs on agricultural soils. However, our study was designed and performed in microcosm laboratory conditions. The performance of AgNPs on the natural ecosystem still needs further study in the future.

## Data Availability Statement

The original contributions presented in the study are included in the article/supplementary material, further inquiries can be directed to the corresponding author.

## Author Contributions

XZ and YY: conceptualization. XZ, DD, LZ, YW, and HL: methodology. XZ, DD, LZ, YW, and YY: formal analysis and investigation. XZ, LZ, and HL: writing-original draft preparation. DD, YW, and YY: writing-review and editing. YY: funding acquisition.

## Funding

The authors acknowledge the financial support from Guangxi Key Science and Technology Innovation Base on Karst Dynamics (no. KDL&Guangxi 202008) and the NUIST Students' Platform for Innovation and Entrepreneurship Training Program.

## Conflict of Interest

The authors declare that the research was conducted in the absence of any commercial or financial relationships that could be construed as a potential conflict of interest.

## Publisher's Note

All claims expressed in this article are solely those of the authors and do not necessarily represent those of their affiliated organizations, or those of the publisher, the editors and the reviewers. Any product that may be evaluated in this article, or claim that may be made by its manufacturer, is not guaranteed or endorsed by the publisher.
